# Late‐Onset Multiple Acyl‐CoA Dehydrogenase Deficiency (MADD): A Case Report With a Complex Biochemical Profile

**DOI:** 10.1002/jmd2.70035

**Published:** 2025-07-18

**Authors:** Romain Penicaud, Jean‐Baptiste Ferron, Xavier Valette, Pierrick Bauduin, Maxime Faisant, Manuel Schiff, Alexandre Nguyen, Fanny Fontaine, Stéphane Allouche

**Affiliations:** ^1^ Department of Clinical Biochemistry Caen University Hospital Caen France; ^2^ Department of Internal Medicine Caen University Hospital Caen France; ^3^ Department of Critical Care Medicine Caen University Hospital Caen France; ^4^ Department of Pathobiology Caen University Hospital Caen France; ^5^ Reference Center for Inborn Errors of Metabolism and Reference Center for Mitochondrial Disorders, Department of Pediatrics, Necker‐Enfants‐Malades Hospital, Assistance Publique‐Hôpitaux de Paris University of Paris‐Cité Paris France; ^6^ Department of Internal Medicine Falaise Hospital Falaise France

**Keywords:** acylcarnitine profile, beta‐oxidation, electron transfer flavoprotein dehydrogenase (ETFDH), mitochondrial disease, multiple acyl‐CoA dehydrogenase deficiency (MADD), rhabdomyolysis

## Abstract

Three clinical entities of multiple acyl‐CoA dehydrogenase deficiency (MADD, OMIM#231680) can be differentiated: two severe neonatal forms and one later‐onset form that can manifest in adulthood. The latter typically presents with muscle‐related symptoms, such as exercise intolerance and muscle weakness, with an increase in all chain‐length acylcarnitine species on the acylcarnitine profile. Here, we report the case of a 33‐year‐old woman who experienced severe psychiatric issues complicated by an eating disorder resulting in a significant malnutrition a few months before presenting with a profound muscle weakness, fatigue, intermittent ptosis, and a major rhabdomyolysis (creatine kinase (CK) > 15 000 IU/L). Biochemical blood tests, including acylcarnitine profile, urinary organic acid analysis, and in vitro beta‐oxidation, were not suggestive of a metabolic disease. Given the absence of an etiology and the worsening health condition of the patient, whole exome sequencing (WES) was performed and revealed two pathogenic variants in the electron transfer flavoprotein dehydrogenase (*ETFDH)* gene, whose association has not been reported before. Unlike other beta oxidation deficiencies, which have a typical biochemical signature, the diagnosis of MADD was made by genetic analyses, which allowed the introduction of vitamin B2 supplementation and the rapid improvement of symptoms.

**Summary**

- This adult case of multiple acyl‐CoA dehydrogenase deficiency (MADD) highlights the diagnostic challenges, emphasizing the need for comprehensive genetic testing in cases of recurrent muscle symptoms and metabolic decompensations, even in adulthood, to prevent severe complications and ensure timely intervention.

## 1 Introduction

**FIGURE 1:**
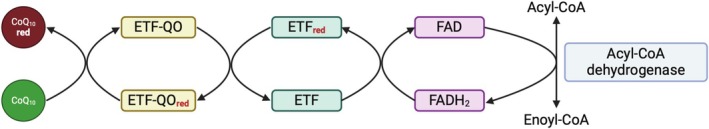
Representation of the electron transport pathway between FAD‐dependent dehydrogenases involved in the beta‐oxidation and the respiratory chain. FAD is reduced to FADH_2_ by dehydrogenases acting on acyl‐CoA of various chain lengths. FADH_2_ is subsequently oxidized by ETF, which in turn is oxidized by ETF‐QO, ultimately transferring the electrons to coenzyme Q_10_ to fuel the respiratory chain. Red: reduced.

The mitochondrial electron transfer flavoprotein ubiquinone oxidoreductase (ETF‐QO) is involved in the electron transport chain linking different dehydrogenases to coenzyme Q_10_. Mutations in the gene (electron transfer flavoprotein dehydrogenase (*ETFDH*)) coding for this protein are responsible for multiple acyl‐CoA dehydrogenase deficiency (MADD), also known as glutaric acidemia type II (GAII) (OMIM#231680), a recessive autosomal metabolic disorder. Two other key players in this process are electron transfer flavoproteins (ETFA and ETFB) which are reduced by flavin adenine dinucleotide (FAD)‐dependent dehydrogenases, including those of the fatty acid beta‐oxidation pathway, as well as others involved in the metabolism of certain amino acids, such as tryptophan and lysine (Figure 1) [1].

These proteins are produced from three genes: *ETFDH*, *ETFA* and *ETFB* that encode the heterodimer ETF. The function of ETF‐QO as well as ETF relies on riboflavin (vitamin B2, the precursor of FAD); therefore, disorders of riboflavin metabolism can result in a secondary deficiency presenting also as MADD. In MADD, mutations in all three genes have been described with a higher prevalence of mutations in *ETFDH* compared to the others (93% for *ETFDH*, 5% for *ETFA*, and 2% for *ETFB*). In the absence of mutations identified in those genes, it is also important to investigate all of the other genes involved in vitamin B2 metabolism, such as genes encoding for riboflavin transporters (*SLC52A1*, *SLC52A2* and *SLC52A3*), genes involved in riboflavin intracellular metabolism (*FLAD1*), or mitochondrial FAD transport (*SLC25A32*) [2].

The clinical presentations of MADD are categorized into three distinct groups. Types I and II are neonatal‐onset forms, with or without congenital malformations at birth, respectively. They are highly severe, with significant mortality, and typically present with episodes of severe hypoglycemia, hypoketotic states, metabolic acidosis, and multiorgan failure. In contrast, type III represents the later‐onset category, characterized predominantly by muscular manifestations such as rhabdomyolysis, myasthenia, and muscle fatigability [3]. Visceral abnormalities have also been reported, including muscular lipidosis and hepatic steatosis, with or without hepatomegaly ([4]; Nana Sede [5]).

Therapeutic management includes a low‐fat, low‐protein diet with increased carbohydrate intake. Carnitine depletion is prevented through l‐carnitine supplementation, and coenzyme Q10 is supplemented if necessary. Prolonged fasting longer than 12 h should be avoided in adults. The most important initial step is to conduct a therapeutic trial with vitamin B2 supplementation, as 98% of late‐onset forms respond to riboflavin supplementation [6].

## 2 Case Presentation and Discussion

A 33‐year‐old Caucasian woman with a long‐standing medical history, including a severe depressive disorder first diagnosed at age 20 (characterized by depression, dissociative psychosis and eating disorders resulting in severe malnutrition), was referred to our neurology department for evaluation of recent‐onset symptoms, including muscle weakness, walking difficulties, fatigue, and intermittent ptosis. She had two recent admissions to the intensive care unit for severe fasting‐induced ketosis in the context of an eating disorder and significant vomiting. During hospitalization, a hepatic steatosis was discovered. Her regular treatments included nutritional supplementation (vitamin B1, B6, B9, B12 and D), an antipsychotic medication (aripiprazole) and pyridostigmine for a suspicion of myasthenia.

Upon admission into the neurology department, the patient's condition deteriorated, presenting with respiratory muscle weakness, head lag, impaired mastication, and dysphagia. At that time, blood glucose measurement revealed mild hypoglycemia at 0.47 g/L (reference value 0.65–1 g/L) with adapted ketosis at 2 mmol/L (reference value < 1 mmol/L). The remaining investigations revealed significantly abnormal results for liver function, with aspartate transaminase (AST) levels exceeding over 100 times the reference value (due to liver and muscle damage) and over 20 times for alanine transaminase (ALT), rhabdomyolysis with creatine kinase (CK) levels at 17 808 IU/L (reference value 20–145 IU/L), anuric renal failure due to acute tubular necrosis and myocardial injury with elevated troponin Ic levels at 671 ng/L (reference value < 15.6 ng/L), without evidence of infarction (cardiac explorations showed pericardial effusion with no sign of ischemic sequelae).

The diagnostic hypotheses considered at that time included an inflammatory myositis, a metabolic myopathy, or Wilson's disease related to neuro‐hepatic involvement. Autoantibody testing for autoimmune myositis was negative, and given that neither the muscle magnetic resonance imaging nor the patient's clinical context clearly supported this diagnosis, it was ruled out. Laboratory parameters for Wilson's disease revealed an increase in the relative exchangeable copper (REC), defined as the ratio of exchangeable copper to total copper. Initial results showed a value of 9.9% (reference value < 18.5%), which increased to 21.2% and 23.8% in subsequent tests following nutritional repletion. Given this unusual evolution of the REC, molecular genetic testing of the *ATP7B* gene was requested but did not reveal any pathogenic variations.

The acylcarnitine profile revealed a moderate decrease in carnitine levels at 7.8 μmol/L (reference value > 10 μmol/L), likely due to nutritional deficiency, and an isolated increase in glutarylcarnitine (C5‐DC) at 1.49 μmol/L (reference value < 0.3 μmol/L). It is worth noting that this analysis was performed during the acute phase, particularly in the context of renal failure, where it is well recognized that there is a decreased excretion of dicarboxylic acids such as C5‐DC [7]. The urinary organic acid chromatography showed an increased excretion of metabolites indicative of organ damage, such as liver injury (4‐hydroxyphenyllactic acid) and lactic acid. Additionally, elevated levels of metabolites suggestive of vitamin B12 deficiency, such as methylmalonic acid, were observed. After specific analysis, 3‐hydroxyglutaric acid or glutaric acid were not detected. Thus, we considered the increase in C5‐DC as secondary. Additionally, in subsequent follow‐ups, the acylcarnitine profile fully normalized after carnitine supplementation and recovery of renal function.

Amino acid profile revealed an isolated and moderate decrease in citrulline levels at 14 μmol/L (reference range: 19–51 μmol/L), potentially indicative of intestinal epithelial damage.

To summarize, the below biochemical investigations were non‐diagnostic for any metabolic disorder, showing secondary and moderate abnormalities.

To explore the hypothesis of a potential mitochondrial disease, a muscle biopsy was performed for histopathological and biochemical analyses. Neither ragged‐red fibers nor scattered cytochrome c oxidase‐negative fibers suggestive of a mitochondrial disease were observed, but only a slight accumulation of lipid droplet in aerobic fibers. It is worth noting that in adult‐onset MADD, muscular lipidosis is a commonly described feature [1]. Mitochondrial function was assessed through the measurement of respiratory chain complexes by spectrophotometry. The results revealed a partial and combined deficiency in Complexes I (38% of the lowest reference value [LRV]), II (59% of LRV), II + III (46% of LRV), and IV (44% of LRV), without abnormalities in Complexes I + III, III, or V. These findings suggested a secondary impairment of the respiratory chain, given the involvement of different respiratory complexes, particularly Complex II, which is encoded only by nuclear genes. We also evaluated the mitochondrial respiration in the patient's fibroblasts using oxygraphy in the presence of different substrates (malate, glutamate, succinate and palmitate), but we were unable to disclose any abnormality. Those results do not support a constitutional deficiency of the respiratory chain but rather a secondary mitochondrial dysfunction. We also carried out a coenzyme Q10 assay on muscle, which showed no decrease.

Subsequently, using whole exome sequencing (WES) we identified two heterozygous variations in the *ETFDH* gene: NM_004453.4:c.413T>G p.(Leu138Arg) and c.1514T>C p.(Ile505Thr). Family segregation revealed that both variants were in trans position. The first variant is a missense mutation, described in ClinVar and GnomAD as pathogenic/likely pathogenic [8], and was reported in association with the variant NM_004453.4:c.1667C>G p.(Pro556Arg) in a young child affected by a neonatal‐onset MADD [9]. This variation is located in the coenzyme Q10‐binding domain, where deleterious mutations were previously reported [10]. A functional study showed that the p.(Leu138Arg) mutation severely impaired enzyme activity and reduced protein expression [8]; interestingly, a high dose of riboflavin was able to significantly but moderately rescue ETF‐QO activity in vitro. The second variant c.1514T>C is classified as likely pathogenic and is located within the iron–sulfur center domain, a region where mutations are exceedingly rare and seldom reported in cases of MADD. To the best of our knowledge, this variation was only reported at the homozygous state in a 35‐year‐old woman who experienced progressive muscle weakness for 2 years and showed clinical improvement under riboflavin supplementation [11]. Whereas no functional study was performed on this variant, abnormalities were observed both on the acylcarnitine profile (increase of C4, C8 and C14:1 acylcarnitine) and on the urinary organic acid chromatography (increased excretion of 2‐hydroxy‐glutaric acid, 3‐hydroxy‐glutaric acid, 2‐hydroxy‐isovaleric acid and ethylmalonic acid); thus, the authors classified this variant as probably pathogenic. Our case is the first to describe the combination of these two variants, the second probably being less deleterious, explaining the late phenotype and the unusual biochemical profile observed here.

The biochemical diagnosis of MADD primarily relies on plasma acylcarnitine profiling and urinary organic acid chromatography. The acylcarnitine profile typically shows an increase in fatty acids ranging from C4 to C18, reflecting the functional deficiency of acyl‐CoA dehydrogenases specific to multiple chain lengths. Additionally, an elevation in glutarylcarnitine (C5‐DC) is observed since the glutaryl‐CoA dehydrogenase, involved in the catabolism of lysine and tryptophan, is a FAD‐dependent enzyme which transfers electrons to ETF. Organic acid chromatography reveals excessive urinary excretion of 2‐hydroxyglutaric acid, 2‐glutaric lactone, glycine derivatives (e.g., hexanoylglycine and suberylglycine) resulting from detoxification of certain fatty acids, and ethylmalonic acid [12] Following the identification of mutations in the *ETFDH* gene, we also carried out in vitro fatty acid oxidation flux from a blood sample, which showed no deficiency compared with controls (not shown).

Notably, adult‐onset forms of MADD are often responsive to riboflavin (vitamin B2) supplementation, leading to significant clinical improvement after treatment initiation [13]. Following the initiation of treatment, including oral riboflavin at high doses (1000 mg/d in four divided dosages), oral l‐carnitine at 2 g/d and oral sodium 3‐hydroxybutyrate (12 g/d in four divided dosages) along with continuous enteral low‐fat nutrition, the patient's clinical condition improved markedly resulting in a significant clinical improvement in muscular function.

Despite the clinical presentation with features typically observed in MADD (rhabdomyolysis and muscle weakness), our patient also exhibited gastrointestinal (vomiting, poor feeding and weight loss) and neuropsychiatric symptoms, previously reported in late‐onset MADD [14], which significantly complicated the diagnostic process. Furthermore, the biochemical abnormalities, notably the increase in glutarylcarnitine observed during the decompensation phase, was rather considered as secondary and likely due to renal failure since they further normalized following recovery of renal function. Additionally, confounding findings during the investigation for Wilson's disease also diverted attention from the primary diagnosis.

After discharge from the intensive care unit, the patient was transferred to a rehabilitation unit where management included maintenance of pharmacological treatment with riboflavin (1 g/day), levocarnitine (2 g/day), and sodium beta‐hydroxybutyrate (12 g/day), in addition to a low‐fat diet. At one‐month post‐ICU discharge, enteral nutrition was maintained exclusively during the night, while oral intake was increased to four meals per day. By the second month, nocturnal enteral feeding was progressively reduced, and raw cornstarch was introduced in the evening to support metabolic stability. At 3 months from diagnosis, the patient exhibited clinical improvement, with increased walking distance. Nighttime enteral nutrition was still ongoing alongside the evening cornstarch supplementation. Due to difficulties with treatment adherence, the riboflavin dose was reduced from 1 g to 600 mg per day. At 4 months, nocturnal enteral nutrition was withdrawn, and only raw cornstarch was maintained in the evening. The patient was discharged from the rehabilitation unit and the patient was followed up on an outpatient basis. At 5 months, the walking distance had increased to approximately 1 km, although the patient continued to report marked fatigability and exertional dyspnea. By the ninth month, no further improvement in walking distance was observed, but exertional dyspnea had diminished.

Regarding the patient's most recent laboratory work‐up, all results were within normal ranges. Notably, CK levels had normalized, and no abnormalities were observed on the chromatographic analysis of organic acids or the plasma acylcarnitine profiles. Liver function tests had fully normalized.

## 3 Conclusion

The biochemical phenotype of adult‐onset MADD appears challenging to interpret, as it is not consistently pathological even during the acute phase, as we illustrated in our case. In other words, a normal acylcarnitine profile or minor abnormalities potentially deemed nonspecific, such as a mild increase in C5DC, do not rule out MADD and should prompt molecular testing as well as systematic trials with high doses of oral riboflavin. As demonstrated in our case, such management, along with avoidance of fasting and a low‐fat diet, was lifesaving with striking clinical improvement. Last but not least, in the setting of psychiatric manifestations often associated with late‐onset MADD presentations and particularly ETFDH, sertraline therapy should be strictly contraindicated, as it was recently suggested to induce an acquired form of MADD in some patients [15, 16]. Therefore, it should not be used in patients with MADD from genetic origins, such as ETFDH. Of note, our patient was never treated with sertraline.

## Author Contributions

**Romain Penicaud:** analysis and interpretation of data, drafting the article. **Jean‐Baptiste Ferron:** clinical management of the patient, revising the manuscript. **Xavier Valette:** clinical management of the patient, revising the manuscript. **Pierrick Bauduin:** clinical management of the patient, revising the manuscript. **Maxime Faisant:** analysis and interpretation of data, revising the manuscript. **Manuel Schiff:** clinical management of the patient, revising the manuscript. **Alexandre Nguyen:** clinical management of the patient, revising the manuscript. **Fanny Fontaine:** analysis and interpretation of data, revising the manuscript. **Stéphane Allouche:** conception, analysis and interpretation of data, drafting the article. All authors have accepted manuscript the submission.

## Ethics Statement

All procedures performed in this study involving human participants were carried out in accordance with the ethical standards of the University Hospital of Caen, and the 1964 Declaration of Helsinki and its later amendments or comparable ethical standards.

## Consent

Informed consent was obtained from the patient.

## Conflicts of Interest

The authors declare no conflicts of interest.

## Data Availability

The data that support the findings of this study are available from the corresponding author upon reasonable request.
